# Hyperacute reactivation of cytomegalovirus-induced gastroduodenitis during remission induction in a young male patient with granulomatosis with polyangiitis: a case report and review of literature

**DOI:** 10.1186/s13256-025-05103-7

**Published:** 2025-02-24

**Authors:** Nisha Annie George, Sandeep Surendran, Roopa Rachel Paulose, Manu Pradeep

**Affiliations:** 1 Department of Infectious Diseases and HIV Medicine, University Hospitals of Leicester NHS Trust, Leicester, UK; 2 Department of Rheumatology and Clinical Immunology, Amrita Institute of Medical Sciences, Amrita Vishwa Vidyapeetham, Kochi, Kerala India; 3 Department of Pathology, Amrita Institute of Medical Sciences, Amrita Vishwa Vidyapeetham, Kochi, Kerala India

**Keywords:** Granulomatosis with polyangiitis, Wegner’s granulomatosis, Cytomegalovirus, Steroids, Gastroduodenitis

## Abstract

**Background:**

Cytomegalovirus is a pathogen known to aggravate the inflammatory response in autoimmune diseases via molecular mimicry. Although it is recognized that cytomegalovirus activation can happen during extended but variable periods of immunosuppression (14–90 days), it is rarely reported in conjunction with an acute flare-up of an autoimmune disease. Currently, there is no consensus on cytomegalovirus prophylaxis for patients initiating remission induction.

**Case presentation:**

Here, we present the case of a 31-year-old male patient of South Indian ethnicity, presenting with a 2-month history of fever, conductive hearing loss, and ear discharge. This was associated with symmetrical inflammatory polyarthritis for 1 month, unilateral painful conjunctivitis, and skin erythema for 5 days. Blood analyses showed elevated inflammatory markers; strongly positive anti-proteinase 3 and cytoplasmic antineutrophil cytoplasmic antibody levels; normal procalcitonin and complement levels; and negative anti-myeloperoxidase and perinuclear antineutrophil cytoplasmic antibody levels. A nasal endoscopy revealed a midline granuloma with vasculitis features on biopsy. Imaging revealed pulmonary nodules and otomastoiditis. Now diagnosed with granulomatosis with polyangiitis, the patient developed signs of gastroduodenitis within a day of initiation of immunosuppression with high-dose “pulse” intravenous methylprednisolone. We evaluated him for mesenteric ischemia/gastrointestinal vasculitis. However, the duodenal biopsies from the bleeding ulcers revealed a probable cytomegalovirus infection, confirmed with high serum viral loads. We treated him with a ganciclovir regimen and transitioned him to steroid-sparing immunosuppressant therapy with mycophenolate mofetil, which was selected over cyclophosphamide for its noninferior effectiveness and better safety profile in non-life-threatening granulomatosis with polyangiitis disease. The patient recovered uneventfully and is currently in remission.

**Conclusion:**

Cytomegalovirus reactivation is possible during short-term steroid pulse therapy. Further research is needed to evaluate whether routine cytomegalovirus screening is warranted before starting immunosuppressive treatment with high-dose steroids in autoimmune conditions.

## Introduction

Cytomegalovirus (CMV), part of the *B-herpes*virus family, contributes to increased morbidity and mortality when reactivation occurs in immunosuppressed individuals [1]. CMV infections range from asymptomatic viremia to systemic manifestations requiring antiviral treatment [2]. Granulomatosis with polyangiitis (GPA) is a form of vasculitis characterized by inflammation of blood vessels, affecting organs such as the lungs and kidneys. Patients with GPA often receive immunosuppressive therapies, including corticosteroids and biologics, which can predispose them to opportunistic infections such as CMV. Studies have shown that CMV can trigger or exacerbate autoimmune responses, potentially leading to disease flares or complications [3]. The interplay between CMV infection and GPA is complex, with evidence suggesting that viral reactivation may coincide with periods of heightened disease activity, complicating management strategies for affected patients [4].

CMV can exacerbate autoimmune diseases through several mechanisms:

- Molecular mimicry: viral antigens may resemble host tissue proteins, leading to cross-reactivity and autoantibody production.
- Cytokine production: CMV infection can induce a proinflammatory cytokine environment, promoting immune dysregulation and autoimmunity.
- Toll-like receptor (TLR) activation: TLRs can be activated by CMV, triggering innate immune responses that may enhance adaptive immunity against self-antigens.

These mechanisms collectively contribute to the development or worsening of autoimmune conditions by fostering an inflammatory milieu that disrupts normal immune tolerance [5, 6].

Despite the recognized association between CMV reactivation and GPA, there remains a significant knowledge gap regarding the specific timelines of CMV reactivation in patients with GPA undergoing pulsed steroid therapy. While current literature suggests that CMV infections are often seen after prolonged immunosuppression, ranging anywhere between 14 and 90 days [7–9], we still lack comprehensive studies on the reliable incidence, risk factors, and timing of CMV reactivation in this population, particularly in relation to treatment regimens. Further research is essential to establish effective CMV screening protocols and prophylactic strategies for patients with GPA receiving immunosuppressive therapies, ensuring better management of potential complications associated with viral reactivation.

We present a case report of a young patient who presented with acute systemic manifestations of granulomatosis with polyangiitis (GPA) and who developed melena secondary to CMV duodenitis immediately after initiation of intravenous steroids in a quaternary care hospital in Kerala, India.

## Case presentation

A 31-year-old male patient of South Indian ethnicity presented with a 2-month history of low-grade, intermittent fever, new-onset bilateral hearing loss, and intermittent ear discharge. This was associated with progressively increasing joint pain and restriction of movement involving bilateral wrists, elbows, shoulders, knees, and ankles for 1 month. He also developed a unilateral red eye with pain, as well as a raised red rash on his legs, for 5 days. We admitted him to the rheumatology department for evaluation. He was moderately built with no past medical history and a family history of primary immunodeficiency. Physical examination revealed bilateral tender and swollen knee, elbow, wrist, and ankle joints with a restricted range of motion. The bilateral lower limb rash was maculopapular.

Pure-tone audiometry confirmed bilateral severe conductive hearing loss with middle ear effusion. Nasal endoscopy indicated a probable midline granulomatous lesion in the nose. Nonenhanced computed tomography (CT) imaging of the chest and high-resolution CT of the temporal bone revealed right mid-zone consolidation with multiple bilateral nodules and oto-mastoiditis, respectively. Laboratory investigations indicated elevated inflammatory markers (erythrocyte sedimentation rate, 90 mm in 1 hour; C-reactive protein, 165 mg/L) and a serum procalcitonin level of 0.44 ng/mL with normal complement levels. Liver function parameters were within normal limits. A rheumatoid factor assay exhibited low titers at 22 IU/mL (normal laboratory range < 18 IU/mL). His cytoplasmic antineutrophil cytoplasmic antibodies (c-ANCA), via immunofluorescence (IF) assay, were positive, with strongly positive anti-proteinase 3 (anti-PR3) antibody titers via enzyme-linked immunosorbent assay (ELISA) (95.01 U/mL) and negative anti-myeloperoxidase (anti-MPO) and perinuclear antineutrophil cytoplasmic antibody (p-ANCA) levels. Urinalysis did not reveal renal involvement. Skin biopsy exhibited vasculitic features, which were negative for IF deposition. Antinuclear antibodies (ANA), from an IF assay, and angiotensin converting enzyme (ACE) were negative. An initial blood culture returned no growth. Thus, per the 2022 American College of Rheumatology (ACR)/European League against Rheumatism (EULAR) criteria, we established a GPA diagnosis with a total score of 12 [3]. The patient did not have any symptoms or signs of tuberculosis, and sputum cultures were negative for acid-fast bacilli. He also screened negative for hepatitis B and C and human immunodeficiency virus (HIV). We do not routinely screen for CMV infections in patients with GPA.

**Fig. 1:**
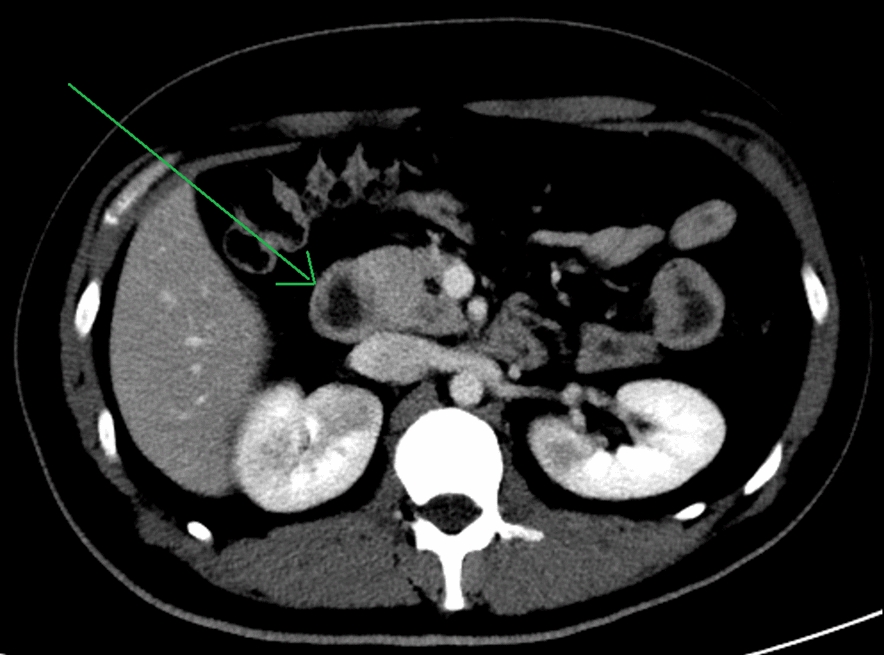
Axial view of abdominal computed tomography angiograph, with the arrow pointing to a 7.1 mm thickening of the second duodenal segment, suggesting duodenitis

As indicated in GPA with pulmonary involvement, he was due to receive a 3-day pulse dose of intravenous methylprednisolone 1 gm/day. While his general condition improved on day 1 after he received the steroid pulse, he developed persistent episodic epigastric abdominal pain on day 2. We considered gastrointestinal vasculitis; however, abdominal CT angiographic imaging revealed no evidence of suggestive mesenteric ischemia/vasculitis. However, we noted duodenal wall thickening—suggestive of nonspecific duodenitis (Fig. 1). Subsequently, 3 days after starting intravenous steroids, he developed melena. Gastroscopy displayed edematous, friable mucosa with scalloping and multiple 3–5 mm clean-based ulcers in the second and third duodenal segments, with only mild spontaneous oozing. In view of gradually decreasing hemoglobin values, we performed a capsule endoscopy to rule out more active bleeding elsewhere—which showed multiple (20+) 1–3 mm small intestinal ulcerations—yet did not reveal any active bleeding. Thus, he was managed conservatively. Then, 3 days later, he developed sudden hypotension with active melena with a drop in hemoglobin values from 8 gm/dL to 5 gm/dL. He was transferred to the intensive care unit (ICU) for stabilization. Suspecting an acute bleed from the duodenal ulcers, we performed an urgent enteroscopy, which showed multiple (20+) duodenal (D3 and D4 segments) and jejunal ulcers of varying sizes (largest 2 cm × 2 cm). Ulcers with active or recent signs of bleeding/pulsating vessels were treated with hemoclips, achieving hemostasis.

A biopsy of the ulcers revealed duodenal-type mucosa with maintained villous architecture, lamina propria cellularity, muscularis mucosa, and scanty submucosa. No neutrophilic activity was noted, nor was there any evidence of small vessel vasculitis. However, the pathologist detected a lone “owl-eye” CMV-like inclusion body in an endothelial cell. CMV immunohistochemistry was inconclusive as the focus of interest was depleted on deeper sections. Given this histopathological finding and the gastrointestinal ulcers, with other vasculitic systemic symptoms resolving in the background, we suspected a CMV infection. Empirically, we initiated intravenous gancyclovir 5 mg/kg/day and other supportive measures in the ICU. We performed a quantitative CMV DNA polymerase chain reaction (PCR) test on the patient’s plasma, which returned a value of 1973 copies/mL (laboratory reference range < 60 copies/mL). He improved clinically and was transitioned to the ward, gradually resuming oral feeds. His serial hemoglobin values remained stable after that.

Systemic vasculitis continued to respond well to treatment, with a resolution of skin lesions, oral lesions, arthritis, and lung consolidation. On repeat pure tone audiometry, bilateral conductive hearing loss improved from severe to moderate, albeit persistent. However, we investigated the patient for an underlying immunodeficiency owing to the rapid CMV flare. Immunoglobulin subset analysis indicated low levels of immunoglobulin G (IgG) (479 mg/dL) and immunoglobulin M (IgM) (25.5 mg/dL). Despite being commenced on steroids, lymphocyte subset analysis showed normal T/B/natural killer (NK) lymphocyte levels. Whole exome sequencing was negative for common variable immunodeficiency (CVID), suggesting the low immunoglobulin levels were likely secondary to a combination of glucocorticoid use and critical illness-related immunosuppression.

**Fig. 2:**
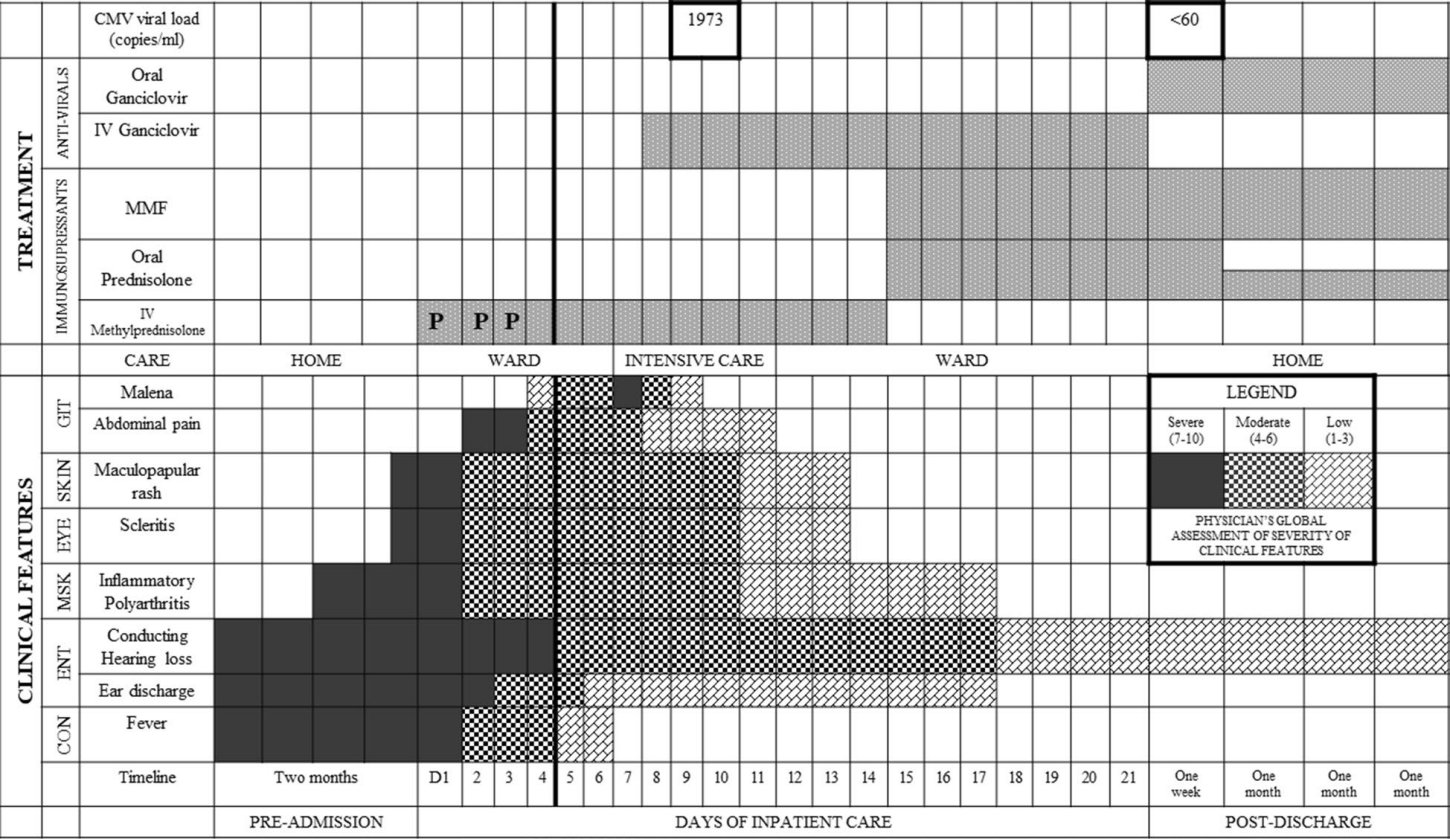
Timeline of system-specific clinical features and treatments provided. The bold line at day 4 of admission depicts the start of gastrointestinal symptoms after the steroid pulse (P) regimen, after other vasculitis symptoms began resolving. These gastrointestinal symptoms started resolving after starting intravenous ganciclovir; *CON* constitutional symptoms, *ENT* ear, nose, and throat system, *MSK* musculoskeletal system, *GIT* gastrointestinal system

For systemic GPA without renal involvement, the patient was prescribed oral prednisolone (1 mg/kg/day) along with mycophenolate mofetil (MMF) as the second-line agent for induction. In the context of CMV duodenitis, we considered MMF to be a noninferior but primarily safer alternative to cyclophosphamide or rituximab [10–13]. For glucocorticoid-induced hypogammaglobulinemia, we administered IgG replacement (0.4 mg/kg/day) as a single dose, and steroids were rapidly tapered [14]. Oral ganciclovir 900 mg every 12 hours was continued for 3 weeks, followed by a 3-month prophylactic dose, which was tapered alongside glucocorticoids. The clinical course and treatment timeline is depicted in Fig. 2. Serial monitoring of immunoglobulin levels showed no further drop. Following the induction phase, rituximab (500 mg every 6 months) was administered for 2 years as part of maintenance therapy, aligned with European Alliance of Associations for Rheumatology (EULAR) recommendations [11]. The institutional review board approved the conduct of this study, and the patient provided written informed consent to publish this case.

## Discussion and conclusion

Hyperacute reactivation of CMV infection in our patient initiated by immunosuppression could be attributed to multiple pathological processes. The initiation of high-dose methylprednisolone could have suppressed the CD4 T lymphocyte function—which is essential for limiting CMV viral replication—leading to a rapid increase in viral load from otherwise dormant CMV virions in monocytic and pre-monocytic cells [15, 16]. The systemic inflammation owing to the GPA disease activity could itself impair natural killer (NK) cell activity and increase the patient’s susceptibility to infections such as CMV [3, 17].

CMV-reactivation, secondary to an initial insult, may prolong and amplify the inflammatory process via increased production of type I cytokines, which can induce autoimmune states [1]. Evidence shows that through molecular mimicry, tissues with CMV proteins participate in autoimmune reactions, which is well studied in systemic sclerosis where CMV RNA was detected around endothelial cells in skin biopsies. It triggers fibrotic and vasculogenic cascades seen in systemic sclerosis. Genetic susceptibility and defective immunity lead to sustained viremia with hyperfunctional toll-like receptor 3 (TLR), which load the gun that triggers immunogenicity and severity in systemic lupus erythematosus (SLE) [18]; similar mechanisms were deduced in CMV colitis and subsequent inflammatory bowel disease (IBD) diagnoses [8]. A study found that patients with GPA receiving high-dose corticosteroids or immunosuppressive agents were at an elevated risk for CMV reactivation [19]. The maximum count of CMV antigenemia was predictive of serious infections and mortality in this population. Patients experiencing CMV reactivation often have worse clinical outcomes, including increased disease activity and complications related to both the autoimmune disease and the viral infection itself [16, 19].

Varani *et al*. described an immunocompetent patient developing GPA secondary to a CMV infection [20]. However, we did not have early CMV serology (IgM) or IgG available to make any deduction in this case. A literature review has shown differing views on this matter. For one, it is difficult to determine which disease process started first, similar to “the chicken or the egg, which came first” debate. Secondly, although there are some studies regarding CMV’s impact on autoimmune diseases discussed above, more research is needed to establish a causal relationship [5].

Our patient developed signs of upper gastrointestinal bleeding (melena) soon after pulsed steroid therapy—during which his systemic vasculitis symptoms improved. This contrasts with the currently published studies. An 8-year retrospective study conducted by Ozaki *et al*. [7] showed that the average time from pulsed glucocorticoid treatment to CMV in the gastrointestinal tract was 55.5 days (range 33–99 days). Table 1 briefly summarizes key studies and messages concerning gastrointestinal CMV infection in patients with autoimmune disorders. It has been speculated that CMV infection can be described on a spectrum from asymptomatic viremia to systemic infection [2]. It is important to note that a CMV antigenemia assay can make it difficult to ascertain its own clinical significance [7, 21, 22]. Jang *et al*. (2009) established the assay’s sensitivity as 54% [confidence interval (CI): 41–68%]. Quantitative real-time PCR is more sensitive [23] and can be used with microscopic detection of intranuclear inclusion bodies. In addition, immunohistochemistry can increase sensitivity to 75% [21]. Outcomes of medical treatment in irritable bowel disease (IBD) with concomitant CMV infection are notably worse than their CMV-negative counterparts [23]. In patients with autoimmunity, where there is discordance in clinical features with classical autoimmune presentations, clinicians should endeavor to rule out a CMV infection, as it is a very protean disease.

**Table 1:** Overview of studies on CMV reactivation in the gastrointestinal system in patients with autoimmune disorders

Author name	Year of publication	Study design	Participant description	Principal findings
Kishore *et al*. [23]	2004	Prospective case series	63 patients with biopsy-proven IBD were screened for CMV infection	15.8% of patients were infected with CMV with no added risk associated with glucocorticoid usage. The duration of immunosuppression was also not noted
Fujita *et al*. [22]	2008	Retrospective case series	Two patients with lupus and one patient with dermatomyositis	Three patients developed CMV infections at 78 days, 80 days, and 28 days, respectively, after the first immunosuppression treatment
Khan *et al*. [30]	2009	Case report	CMV colitis and newly diagnosed case of lupus nephritis	19-year-old man developed CMW colitis 90 days after initiation of immunosuppression
Ozaki *et al*. [7]	2013	Retrospective case series (2004–2012)	Ten patients with autoimmune disease with upper gastrointestinal CMV infection	The mean interval from initiation of pulsed glucocorticoid therapy (> 250 mg/day) to development of CMV infection was 55.5 days (33–99 days)
Khan *et al*. [8]	2016	Case report	Coexistent CMV colitis and newly diagnosed IBD in an immune-competent man	Isolated CMV colitis in an immunocompetent young patient tends to resolve with minimal complications
Parals *et al*. [31]	2018	Retrospective case series	46 patients with UC having biopsy-proven CMV infections	70% of these patients were on systemic steroids, but the dose and duration of therapy were not available
Morishita *et al*. [32]	2019	Retrospective case series	113 consecutive patients of AAV were analysed for the development of CMV infection	11.7% of patients with AAV developed CMV infection, and it was noted that patients having a severe form of AAV was an independent risk factor for CMV infection
Yeh *et al*. [9]	2021	Retrospective cohort study	42 patients without autoimmunity with infection-associated enteritis (CMV versus non-CMV)	Usage of ≥ 20 mg/day of prednisolone for more than 2 weeks, along with advanced age, was found to be a risk factor for CMV enteritis

*IBD* inflammatory bowel disease, *UC* ulcerative colitis, *CMV* cytomegalovirus, *AAV* antineutrophil cytoplasmic antibody-associated vasculitis

Diagnosing CMV infection in patients with autoimmunity presents significant challenges owing to overlapping symptoms and the immunocompromised state of these individuals. The clinical manifestations of CMV can mimic those of autoimmune diseases, such as fever, fatigue, and organ dysfunction, complicating accurate diagnosis. Furthermore, standard diagnostic tests such as CMV antigenemia and plasma viral load can yield inconsistent results, particularly in patients receiving immunosuppressive therapy, which may alter immune responses and test reliability [19, 24]. Considering CMV as a differential diagnosis is crucial because its reactivation can lead to severe complications, including increased mortality in patients with autoimmunity. Early identification and treatment of CMV can improve patient outcomes, highlighting the need for heightened awareness among clinicians managing autoimmune diseases [25, 26].

Treatment and prophylaxis strategies for CMV in patients with rheumatologic conditions include antiviral therapy, preemptive treatment, and routine monitoring. Pros of antiviral therapy, such as ganciclovir and valganciclovir, include effective viral suppression and reduced morbidity associated with CMV reactivation. Preemptive strategies can lower the incidence of severe infections when implemented early. However, the cons involve potential toxicity, particularly bone marrow suppression, which is critical in immunocompromised patients. In addition, the lack of consensus on optimal monitoring protocols complicates management. Research indicates that adherence to guidelines such as the CMV Infection Preemptive Inpatient Management Guideline (CMVi-PMG) can reduce infection rates, but adherence remains low in practice [27, 28]. There is a pressing need for further studies to establish clear, evidence-based guidelines for CMV management in this population to balance effective prevention with the risks of treatment [16]. In our patient, we administered intravenous gancyclovir for 21 days, after which their CMV viral load was resolved. However, in the context of critical illness/steroid-induced secondary immunosuppression, we continued the oral gancyclovir for 3 months, until the steroid dose was tapered to 7.5 mg/day—along with an initial single dose of immunoglobulin replacement therapy to counteract secondary immunosuppression.

CMV infection is an important cause of mortality and morbidity in patients with autoimmune disorders undergoing immunosuppression. However, unlike transplant recipients, prophylactic antiviral treatments are not standard practice [29]. It is difficult to establish whether prophylactic treatment would be beneficial, as it is routinely done with bone marrow or organ transplant recipients. Further studies will need to be done in this area. While CMV can infect various organ systems in these patients, gastrointestinal infections are significant complications that treating rheumatologists should actively monitor, even in the initial phase of immunosuppressive therapy (Table 1).

Inferences from this case report may be limited by the lack of baseline CMV serology and the possibility of other unknown contributing factors related to the patient’s gastrointestinal symptoms. However, a detailed clinical history of our young patient did not reveal any past symptoms or signs of inflammatory bowel diseases, as well as any history of immunosuppression/immune deficiency. Thus, we reasonably believe that the high-dose steroid immunosuppression, combined with the new-onset GPA disease activity, was the causal trigger for CMV infection.

The present case study reveals that CMV reactivation is possible within hours of high-dose steroid remission induction in patients with active GPA, contrary to current literature. In cases of discordant symptoms in autoimmune disease flare episodes in patients initiated on immunosuppressive therapy, clinicians must consider a developing CMV infection as a differential diagnosis. Early recognition and appropriate management of CMV infection can potentially reduce the morbidity and mortality in such patients. Future studies should investigate optimal strategies for screening, prophylaxis, and treatment in immunosuppression-induced CMV infections in patients with autoimmune diseases.

## Data Availability

Data sharing is not applicable to this article as no datasets were generated or analyzed during the current study.

## Abbreviations

CMV: Cytomegalovirus
GPA: Granulomatosis with polyangiitis
SLE: Systemic lupus erythematosus
IV: Intravenous
IF: Immunofluorescence
CT: Computed tomography
PCR: Polymerase chain reaction
IBD: Inflammatory bowel disease
